# A Decoy Peptide Targeted to Protein Phosphatase 1 Attenuates Degradation of SERCA2a in Vascular Smooth Muscle Cells

**DOI:** 10.1371/journal.pone.0165569

**Published:** 2016-10-28

**Authors:** Seung Pil Jang, Jae Gyun Oh, Dong Hoon Kang, Ju Young Kang, Sang Won Kang, Roger J. Hajjar, Woo Jin Park

**Affiliations:** 1 College of Life Sciences, Gwangju Institute of Science and Technology, Gwangju, Korea; 2 Cardiovascular Research Center, Icahn School of Medicine at Mount Sinai, New York, New York 10029, United States of America; 3 Department of Life Science, Ewha Womans University, Seoul, Korea; Albany Medical College, UNITED STATES

## Abstract

Neointimal growth in the injured vasculature is largely facilitated by the proliferation of vascular smooth muscle cells (VSMC), which associates with reduced sarco/endoplasmic reticulum Ca^2+^-ATPase (SERCA2a) activity. The gene transfer-mediated restoration of the SERCA2a level thus attenuates neointimal growth and VSMC proliferation. We previously reported that a peptide targeted to protein phosphatase 1, ψPLB-SE, normalizes SERCA2a activity in cardiomyocytes. In this study, we found that ψPLB-SE attenuated neointimal growth in balloon-injured rat carotid arteries, and the proliferation and migration of VSMC cultured in high-serum media (synthetic conditions). In parallel, ψPLB-SE inhibited the degradation of SERCA2a in the injured carotid arteries and VSMC under synthetic conditions. The calpain inhibitor MDL28170 also attenuated SERCA2a degradation and VSMC proliferation under synthetic conditions, indicating that calpain degrades SERCA2a. The Ca^2+^ ionophore A23187 induced SERCA2a degradation in VSMC, which was blocked by either ψPLB-SE or MDL28170. Additionally, ψPLB-SE normalized the cytosolic Ca^2+^ level in VSMC that was increased by either A23187 or synthetic stimulation. Collectively, these data indicate that ψPLB-SE corrects the abnormal Ca^2+^ handling by activating SERCA2a, which further protects SERCA2a from calpain-dependent degradation in VSMC. We conclude that ψPLB-SE may form the basis of a therapeutic strategy for vascular proliferative disorders.

## Introduction

The abnormal proliferation of vascular smooth muscle cells (VSMC) is an underlying cause in the pathogenesis of several vascular proliferative disorders such as atherosclerosis and aortic restenosis [1, 2]. When the arterial wall is damaged, VSMC migrate into the intimal layer of the arterial wall and undergo drastic changes in their phenotype from contractile and quiescent to synthetic and proliferative. The uncontrolled proliferation of VSMC with a synthetic phenotype then results in the enlargement of the arterial intima, a phenomenon called neointimal growth [3–5]. Therefore, the modulation of VSMC proliferation is important in the treatment of vascular proliferative disorders.

The proliferation of VSMC associates with a chronic increase in the cytosolic Ca^2+^ level, which is caused by the loss of Ca^2+^ handling proteins such as ryanodine receptors and sarco/endoplasmic reticulum (SR) Ca^2+^-ATPase (SERCA2a) [6]. The gene transfer-mediated restoration of the SERCA2a level attenuates VSMC proliferation and neointimal formation [7–9]. Therefore, the maintenance of a low cytosolic Ca^2+^ level by controlling SERCA2a activity may be a reasonable strategy to prevent VSMC proliferation.

SERCA2a activity is inhibited by a direct interaction with phospholamban (PLB) [10, 11], whose inhibitory activity is enhanced by dephosphorylation at Ser16 or Thr17 by protein phosphatase 1 (PP1) [12–15]. Therefore, the inhibition of the PP1-mediated dephosphorylation of PLB is a reasonable approach to upregulate SERCA2a activity in failing hearts. We previously showed that a 9-mer peptide, ψPLB-SE, mimics phosphorylated PLB, and thus functions as a decoy for PP1 [16]. This peptide restored SERCA2a activity in the heart and improved recovery after ischemia/reperfusion by inhibiting the dephosphorylation of PLB *in vitro* and *ex vivo*.

In this study, we show that ψPLB-SE attenuates neointimal growth in the rat carotid artery by inhibiting the degradation of SERCA2a in VSMC. Under pathological conditions, the increased cytosolic Ca^2+^ level leads to the activation of calpain that is in turn responsible for the degradation of SERCA2a, which further increases the cytosolic Ca^2+^ level. Our data indicate that ψPLB-SE interrupts this vicious cycle of an increase in the Ca^2+^ level and a decrease in the SERCA2a level.

## Materials and Methods

### Ethics statement

Animal experiments using Sprague-Dawley rats were granted by approval of the Institutional Animal Care and Use Committee (IACUC) of Ewha Womans University and conformed to the Guide for Care and Use of Laboratory Animals published by the US National Institutes of Health (The National Academies Press, 8th Edition, 2011). All surgical procedures were performed under inhalational anesthesia with isoflurane gas (N2O:O2/70%:30%), and all efforts were made to minimize suffering. The ten-week-old male Sprague-Dawley rats were obtained from Charles River and used for all *in vivo* experiments. After surgical procedure, rats were monitored in every other day to check whether any adverse events were occurred. Environmental conditions were controlled to provide a temperature of 25 *±* 2°C, a relative humidity of 50 *±* 5% and a 12:12 h light/dark cycle. At the end of experiments, rats were anaesthetized by inhalation of isoflurane gas (N2O:O2/70%:30%) and perfused with heparinized saline, and then carotid arteries and thoracic aorta were removed.

### Chemicals and the synthesis of the decoy peptide

The decoy peptide (RAE^16^TIEMPQ) was derived from the PLB protein sequence surrounding the Ser16 phosphorylation site. To facilitate uptake into cells, the decoy peptide was conjugated to the cell penetrating peptide TAT (YGRKKRRQRRR). The peptides used in this study were ΨPLB-SE and RAETIEMPQ. The peptides (purity, >95%; AnyGen, Gwangju, Korea) were resuspended in double-distilled water at a stock concentration of 3 mM. RASMC and HCSMC were treated with the peptides at a final concentration of 3 μM for 1 h. The calcium ionophore A23187, the calpain I and II inhibitor MDL28170, cycloheximide were purchased from Sigma—Aldrich (St. Louis, MO, USA).

### Balloon-induced injury of the rat carotid artery

The left common carotid artery was injured with an infiltrated 2F Fogarty balloon embolectomy catheter. In brief, the rats were anesthetized with isoflurane gas (70% N_2_O/30% O_2_), the left external carotid artery was exposed, and its branches were electro-coagulated. A catheter was inserted approximately 1 cm into the external carotid artery via a transverse arteriotomy, and endothelial denudation was achieved by three passes of the catheter along the common carotid artery. After removal of the catheter, the penetrated area was clamped, and 5 μg of the peptides solubilized in 200 μL of phosphate-buffered saline (PBS) was injected. After incubation for 15 min, the sealed carotid artery was re-opened to resume blood flow. The rats were allowed to recover for 10 days, unless otherwise stated. For histological analyses, the rats were anesthetized, and the common carotid artery was excised after transcardiac perfusion with heparinized saline containing 3.7% (w/v) formaldehyde. The specimens were embedded in paraffin, and paraffin blocks were sectioned with a Leica RM2255 rotary microtome. Two serial tissue sections (thickness, 4 μm) were obtained from the centre of the common carotid artery and stained with haematoxylin and eosin. The lamina, internal elastic lamina, and external elastic lamina were measured with the National Institutes of Health ImageJ software (version 1.62). The intimal and medial areas were determined by subtracting the laminal area from the internal elastic laminal area and by subtracting the internal elastic laminal area from the external elastic laminal area, respectively. The values from two serial sections per rat were averaged for analysis.

### Immunohistochemistry

The carotid artery was fixed in 4% (w/v) paraformaldehyde for 48 h at room temperature and then washed in PBS. After embedding the specimens in paraffin and sectioning the tissue blocks, the sections were treated with hydrogen peroxide to quench endogenous peroxidase activity, followed by boiling in antigen retrieval buffer. The sections were immunostained with antibodies against α-SMA (Sigma—Aldrich), SERCA2a, SERCA2b (21^st^ Century Biochemical), and PCNA (Abcam).

### Cell culture

RASMC were isolated from the medial layer of the thoracic aorta derived from male Sprague-Dawley rats (body weight, 180–200 g) by enzymatic digestion with collagenase (50 U/mL, Worthington) and pancreatic elastase (0.25 mg/mL, Sigma—Aldrich) for 4 h at 37°C. The cells were collected and resuspended in DMEM containing 20% (v/v) fetal bovine serum. They were then plated on collagen I (Sigma—Aldrich)-coated glass coverslips and incubated at 37°C in an atmosphere of 5% (v/v) CO_2_ and 95% (v/v) air. HCSMC were purchased from Lonza and cultured in SmBM (Lonza) supplemented with 0.5 mg/mL hEGF, 5 mg/mL insulin, 1 mg/mL hFGF, 50 mg/mL gentamicin/amphotericin-B, and 5% fetal bovine serum. HCSMCs were cultured at 37°C in an atmosphere of 5% (v/v) CO_2_ and 95% (v/v) air. RASMC and HCSMC were plated at the densities of 1 x 10^4^ cells/cm^2^ and 1 x 10^5^ cells/cm^2^ for immunostaining and western blotting experiments, respectively.

### Fluorescent immunostaining

RASMC were fixed in 4% (w/v) paraformaldehyde for 10 min at room temperature, washed with PBS, and permeabilized with 0.1% (v/v) Triton X-100 in PBS for 40 min. After washing and blocking with 3% (w/v) BSA in PBS, the cells were incubated overnight at room temperature with antibodies against SERCA2a (1:250), SERCA2b (1:250), and PCNA (1:50) at the mentioned dilutions. On the following day, the cells were washed with PBS and incubated for 1 h at room temperature with anti-rabbit and anti-mouse IgG conjugated to Alexa Fluor 488 and 594, respectively. The cells were mounted with FluoroGuard antifade reagent (Bio-Rad, Hercules, CA, USA), and the coverslips were examined under a fluorescent microscope.

### Proliferation assay

RASMC (1 × 10^4^ cells/well) were seeded on a 96-well microplate and treated with peptides at a final concentration of 3 μM for 24 h. Cell proliferation assays were performed using the EZ-CyTox Cell Viability Assay Kit (Daeil Lab Services Co., Ltd.).

### Aorta *ex vivo* organ culture

A rat thoracic aorta was harvested and incubated in RPMI-1640 medium containing 20 mM HEPES, 2 mM L-glutamine, 100 IU/mL penicillin, and 100 μg/mL streptomycin. The adventitia was removed, and the aorta was cut longitudinally and pinned out onto a set resin. To investigate the degradation of SERCA2a, tissue fragments were cultured for 7–10 days in RPMI-1640 medium containing 20% (v/v) FCS. The medium was replaced every 48 h.

### Western blotting

VSMC were homogenized in a minimal volume of 50 mM Tris-HCl, pH 7.4, supplemented with a broad-spectrum protease inhibitor cocktail (Calbiochem). The proteins were separated by SDS-PAGE and then transferred to polyvinylidene fluoride membranes (Schleicher & Schuell). After blocking with 5% (w/v) non-fat milk for 1 h and washing with TBST, the membranes were incubated with antibodies against SERCA2a, SERCA2b, PCNA, and GAPDH (Sigma—Aldrich). The membranes were subsequently incubated with horseradish peroxidase-conjugated secondary antibodies (Jackson ImmunoResearch, West Grove, PA, USA) and developed using a chemiluminescent substrate (Dogen). The blots were scanned and quantified using LAS software.

### Intracellular Ca^2+^ measurements

The intracellular Ca^2+^ level was measured in VSMC by loading cells with 0.5 μM Fura2-AM (Molecular Probes, Eugene OR, USA), a Ca^2+^-sensitive indicator, for 15 min at 37°C. The fluorescence was recorded using the IonOptix calcium imaging system. VSMC were exposed to light emitted by a 75 W halogen lamp through either a 340 or 380 nm filter while being field-stimulated as described above. The fluorescent emissions were detected between 480 and 520 nm by a photomultiplier tube after an initial illumination at 340 nm for 0.5 s and then at 380 nm for the duration of the recording protocol. The 340 nm excitation scan was repeated at the end of the protocol, and qualitative changes in the intracellular Ca^2+^ level were inferred from the ratio of the Fura2 fluorescent intensity at both wavelengths.

### *In vitro* cell scratch assay

RASMC isolated from the thoracic aorta were cultured in DMEM supplemented with 0.1% (v/v) FBS (Contractile) or in DMEM supplemented with 10% (v/v) FBS (Synthetic) in the presence of 3 μM of control or ψPLB-SE peptide. Alternatively, RASMC cultured in DMEM supplemented with 0.1% (v/v) FBS were treated with 10 ng/ml of PDGF-BB (R&D Systems) (Synthetic) in the presence of 3 μM of control or ψPLB-SE peptide. A region of the RASMC layer was removed by scratching the plate with a sterile 200 μL pipette tip. The cells were incubated for 12 or 24 h and then observed under an IX80 microscope (Olympus). The distance travelled by the cells was measured using MetaMorph software.

### Assay for SERCA2a Stability

RASMC were cultured in DMEM supplemented with 0.1% (v/v) FBS (Contractile) or in DMEM supplemented with 10% (v/v) FBS (Synthetic) in the presence of 3 μM of control or ψPLB-SE peptide. Cycloheximide (Sigma) was added to media to a final concentration of 5 μg/ml. Cells were harvested after 0, 3, and 5 days of incubation and their protein extracts were subjected to western blotting.

### Statistical analysis

All data are reported as the means ± SD. Statistical significance was determined by Student’s *t*-test or one-way ANOVA with Bonferroni post-hoc analysis using StatView 5.0 software (SAS Institute, Cary, NC, USA). A *p*-value <0.05 was considered statistically significant.

## Results

### ψPLB-SE inhibits the proliferation of VSMC

**Fig 1:**
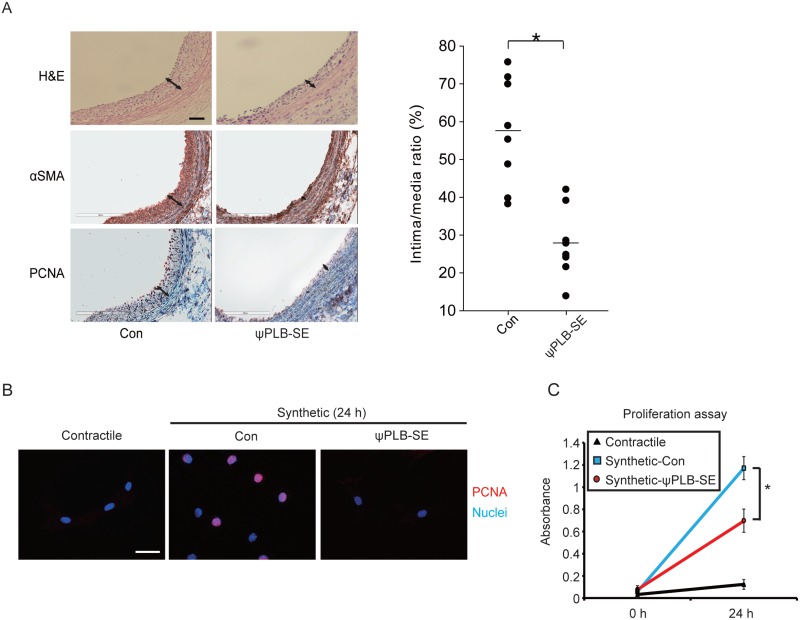
ψPLB-SE attenuates neointimal formation in a model of balloon-induced injury to the carotid artery and RASMC proliferation. The rat carotid artery was subjected to catheter-induced balloon injury. The injured region was treated with 5 μg of ψPLB-SE or control (Con) peptide for 30 min. (**A**) Carotid arteries were sectioned and stained with haematoxylin and eosin (top panels), or immunostained with antibodies against α-SMA (middle panels) and PCNA (bottom panels) 10 days after treatment. The intima/media ratio was calculated (n = 8 each). Scale bar, 50 μm. (**B**) RASMC isolated from the thoracic aorta were incubated in DMEM supplemented with 0.1% (v/v) FBS for 5 days to induce a contractile phenotype, followed by incubation in DMEM supplemented with 10% (v/v) FBS for 24 h to induce a synthetic phenotype in the presence of 3 μM ψPLB-SE or control peptide. Immunostaining was performed with an antibody against PCNA (red). Nuclei were stained with Hoechst (blue). Representative merged images are shown. Scale bar, 50 μm. (**C**) Cell proliferation was quantified using a cell viability assay kit (n = 6).

We previously reported the design of a 9-mer peptide, ψPLB-SE, that improves cardiomyocyte contractility by preserving SERCA2a activity during ischemia-reperfusion injury [16]. In this study, we determined whether this peptide can also inhibit VSMC proliferation through a similar molecular mechanism. An injury in the rat carotid artery was induced by balloon angioplasty, and then ψPLB-SE or control peptide was administered to the injured area. The arteries were harvested 4 weeks after treatment, and tissue sections were subjected to haematoxylin and eosin staining and immunocytochemistry. Haematoxylin and eosin staining revealed that neointimal formation was inhibited in arteries treated with ψPLB-SE compared with those treated with the control peptide. The intimal layer affected by ψPLB-SE was positively immunostained with antibodies against α-smooth muscle actin (α-SMA) and proliferating cell nuclear antigen (PCNA). These data indicate that ψPLB-SE inhibits neointimal growth and VSMC proliferation *in vivo* (Fig 1A).

We then examined the effects of ψPLB-SE on rat aortic smooth muscle cells (RASMC). RASMC exhibited a contractile phenotype when cultured in medium supplemented with a low concentration of serum (0.1% FBS), whereas they adopted a synthetic phenotype in medium with a high concentration of serum (10% FBS). We therefore defined the high serum culture conditions as synthetic in nature. RASMC underwent active cell division under synthetic conditions, as shown by prominent PCNA expression, but this was completely blocked by ψPLB-SE (Fig 1B). Cell proliferation assays also showed that the increased proliferation of RASMC under synthetic conditions was halted by ψPLB-SE (Fig 1C). The increased migratory activity of RASMC under synthetic conditions or upon the treatment of PDGF-BB was also impeded by ψPLB-SE (S1 Fig). These data indicate that ψPLB-SE markedly inhibits proliferation of VSMC under synthetic conditions *in vitro*.

### ψPLB-SE prevents degradation of SERCA2a in VSMC

**Fig 2:**
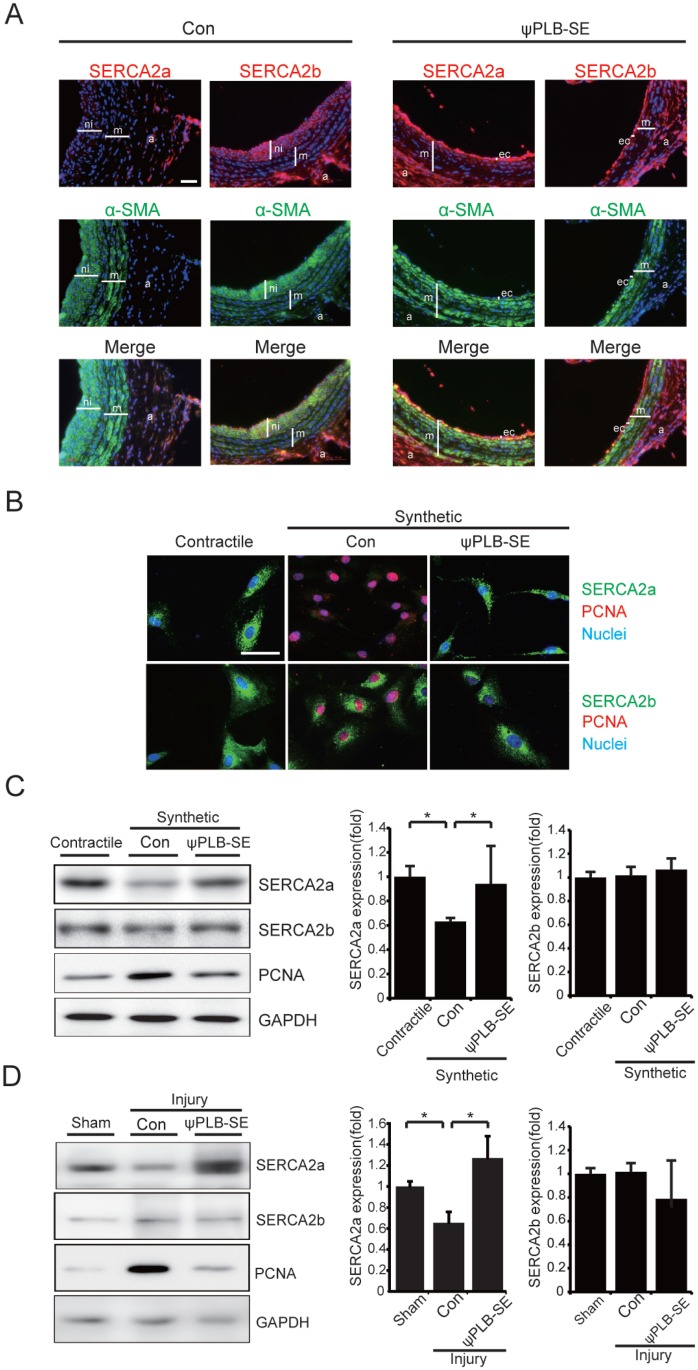
ψPLB-SE prevents degradation of SERCA2a. (**A**) Carotid arteries were sectioned and immunostained with antibodies against SERCA2a or SERCA2b (red) and α-SMA (green) 10 days after peptide treatment. Merged images are shown (bottom). a, adventitia; ec, endothelial cell layer; m, medial layer; ni, neointimal layer. Scale bar, 50 μm. (**B**) RASMC isolated from the thoracic aorta were incubated in DMEM supplemented with 0.1% (v/v) FBS for 5 days to induce a contractile phenotype, followed by incubation in DMEM supplemented with 10% (v/v) FBS for 5 days to induce a synthetic phenotype in the presence of 3 μM ψPLB-SE or control peptide. Immunostaining was performed with antibodies against SERCA2a or SERCA2b (green) and PCNA (red). Nuclei were stained with Hoechst (blue). Representative merged images are shown. Scale bar, 50 μm. (**C**) Western blot analysis performed with cell extracts. (**D**) *Ex vivo* cultures of the thoracic aorta treated with ψPLB-SE or control peptide. Tissue extracts were subjected to western blotting. Data are reported as the means ± SD (n = 3–4; *, *P* < 0.05).

SERCA2a is rapidly degraded in proliferative VSMC, whereas SERCA2b is relatively stable [6]. The restoration of the SERCA2a level by gene delivery inhibits proliferation of VSMC under synthetic conditions. We thus reasoned that the anti-proliferative effects of ψPLB-SE may be associated with the maintenance of the SERCA2a level and activity under synthetic conditions. The carotid artery sections obtained from the experiments described in Fig 1A were subjected to immunostaining with antibodies against SERCA2a, SERCA2b, and α-SMA. While the SERCA2b protein level remained unchanged, the SERCA2a level was significantly reduced upon balloon angioplasty-mediated injury, which was inhibited by ψPLB-SE (Fig 2A). In addition, immunostaining revealed that the SERCA2a level decreased in RASMC under synthetic conditions, and that this SERCA2a reduction was completely inhibited by ψPLB-SE (Fig 2B). Western blotting further confirmed that ψPLB-SE inhibited the reduction of SERCA2a in RASMC under synthetic conditions (Fig 2C). We measured the SERCA2a level in RASMC under conditions where *de novo* protein synthesis was blocked with cycloheximide. The stability of SERCA2a was significantly reduced under synthetic conditions, and it was restored nearly to the level observed under contractile conditions by ψPLB-SE (S2 Fig). An *ex vivo* culture of the rat thoracic aorta was prepared. After the induction of injury by scratching, VSMC exhibited a reduced SERCA2a level, implying the acquisition of a synthetic phenotype. This injury-dependent reduction of SERCA2a in VSMC was completely inhibited by ψPLB-SE (Fig 2D). Collectively, these data indicate that ψPLB-SE inhibits degradation of SERCA2a in VSMC.

### Calpain is responsible for the degradation of SERCA2a in VSMC

**Fig 3:**
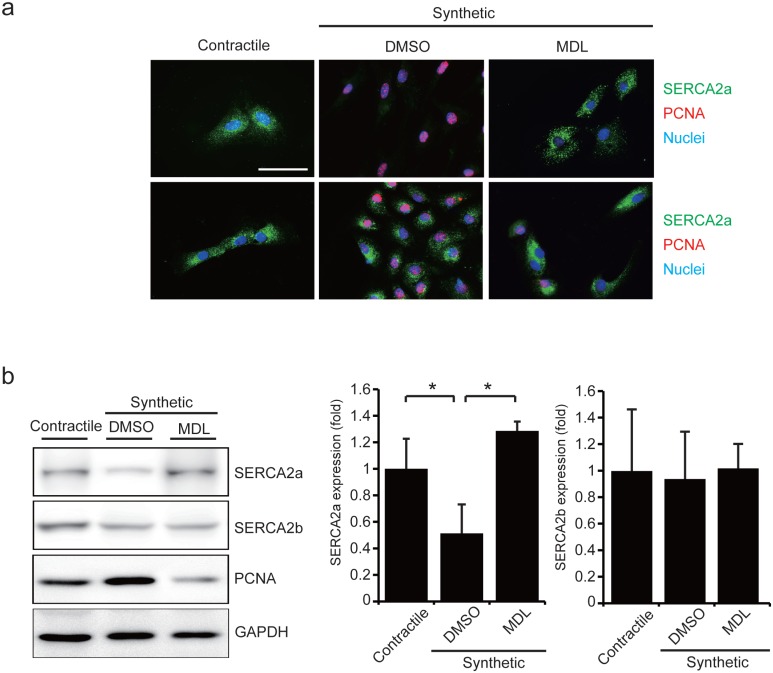
Calpain inhibitor prevents degradation of SERCA2a. (**A**) RASMC were treated with the calpain inhibitor MDL28170 or dimethyl sulfoxide (DMSO) under the same conditions as described for Fig 2B. Immunostaining was performed with antibodies against SERCA2a or SERCA2b (green) and PCNA (red). Nuclei were stained with Hoechst (blue). Representative merged images are shown. Scale bar, 50 μm. (**B**) Western blot analysis performed with cell extracts. Data are reported as the means ± SD (n = 3–4; *, *P* < 0.05).

An increase in the cytosolic Ca^2+^ level induces calpain-mediated degradation of cytosolic proteins [17–19]. Due to the involvement of calpain in the proliferation of VSMC [20, 21], we examined the role of calpain in the degradation of SERCA2a in VSMC. As shown by immunostaining, the calpain inhibitor MDL28170 inhibited the degradation of SERCA2a in RASMC under synthetic conditions (Fig 3A). The protective effect of MDL28170 was further confirmed by western blotting (Fig 3B). These data indicate that calpain mediates the degradation of SERCA2a in VSMC under synthetic conditions. The reduced PCNA level in the presence of MDL28170 also suggests that the sustained SERCA2a level is responsible for the inhibition of VSMC proliferation (Fig 3A).

### ψPLB-SE inhibits calpain-dependent degradation of SERCA2a

**Fig 4:**
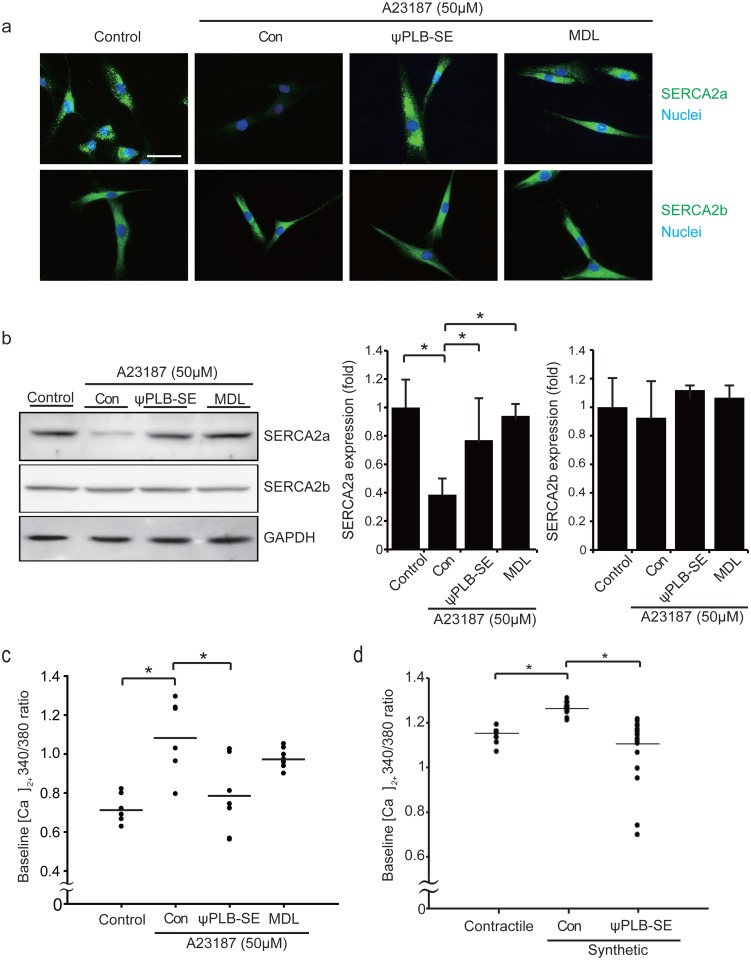
ψPLB-SE inhibits calpain-dependent degradation of SERCA2a. (**A-C**) HCSMC were pretreated for 1 h with 3 μM ψPLB-SE and 15 μM MDL28170, and then treated with 50 μM A23187 for 2 h. (**A**) Immunostaining was performed with antibodies against SERCA2a and SERCA2b (green). Nuclei were stained with Hoechst (blue). Representative merged images are shown. Scale bar, 50 μm. (**B**) Western blot analysis was performed with cell extracts. Data are reported as the means ± SD (n = 3–4; *, *P* < 0.05). (**C**) Baseline Ca^2+^ concentration was measured by IonOptix calcium imaging system. (**D**) RASMC were treated with ψPLB-SE or control peptide under the same conditions as described for Fig 1B. The baseline Ca^2+^ concentration was measured by IonOptix calcium imaging system (*, *P* < 0.05).

The Ca^2+^ ionophore A23187 evoked degradation of SERCA2a, but not SERCA2b, in human coronary smooth muscle cells (HCSMC), indicating that the increased cytosolic Ca^2+^ level triggers the degradation of SERCA2a. The effect of A23187 was completely blocked by MDL28170, revealing that calpain is involved in the degradation of SERCA2a in VSMC upon increase of the cytosolic Ca^2+^ level (Fig 4A and 4B). The Ca^2+^-dependent degradation of SERCA2a was also completely inhibited by ψPLB-SE (Fig 4A and 4B). The A23187-induced elevation of the cytosolic Ca^2+^ level was normalized by ψPLB-SE, but not by the calpain inhibitor (Fig 4C). The cytosolic Ca^2+^ level, which was also elevated under synthetic conditions, was decreased by ψPLB-SE (Fig 4D). Taken together, these data suggest that ψPLB-SE inhibits the calpain-mediated degradation of SERCA2a by normalizing the elevated cytosolic Ca^2+^ level in VSMC under synthetic conditions.

## Discussion

Abnormal intracellular Ca^2+^ handling resulting from a defect in SR function is a leading cause of cardiovascular disease [22–25]. Impaired Ca^2+^ uptake by the SR is mainly due to a decrease in SERCA2a activity that can be caused by changes in its expression and/or post-translational modification [26–28]. The gene transfer-mediated restoration of the SERCA2a level in cardiomyocytes is cardioprotective in mouse and pig models of heart failure [29–32]. In addition, SERCA2a gene transfer inhibits VSMC proliferation and neointimal growth in balloon-induced injury models [7, 8]. Similarly, SERCA2a gene transfer decreases the proliferation and migration of pulmonary artery smooth muscle cells and increases endothelial nitric oxide synthase expression and activation [33] in pulmonary artery endothelial cells. In a monocrotaline-induced pulmonary arterial hypertension (PAH) model, SERCA2a gene transfer normalizes pulmonary artery pressure, vascular remodeling, and fibrosis [34]. Therefore, SERCA2a may offer a therapeutic intervention for vascular proliferative diseases, including arterial restenosis and PAH.

SERCA2a activity is modulated by a series of signaling cascades that involve inhibitor-1 (I-1), PP1, and phospholamban (PLB). I-1 binds and inhibits PP1, resulting in the elevation of PLB phosphorylation and SERCA2a activity. Therefore, the overexpression of constitutively active I-1 (I-1c) can restore SERCA2a activity in cardiomyocytes under various pathological insults [35]. In addition, I-1c gene transfer inhibits VSMC proliferation and neointimal formation by increasing SERCA2a activity. I-1c and SERCA2a gene transfer is synergistically beneficial [36].

We previously showed that ψPLB-SE, a 9-mer peptide, targets PP1 and inhibits PLB dephosphorylation, leading to an increase in SERCA2a activity in cardiomyocytes [16]. Similarly, ψPLB-SE inhibited the dephosphorylation of PLB in RASMC (S3 Fig). In this study, we showed that ψPLB-SE inhibited VSMC proliferation and neointimal formation by elevating the SERCA2a level in VSMC under synthetic conditions. In previous studies, SERCA gene transfer restored the SERCA2a level, whereas I-1c gene transfer restored SERCA2a activity. Therefore, it was surprising that the targeting of PP1 by ψPLB-SE also restored the SERCA2a level. To explain these observations, we propose a model in which the SERCA2a level and activity are tightly interrelated. Under synthetic conditions, the increased cytosolic Ca^2+^ level may activate calpain to degrade the SERCA2a protein, and the decreased SERCA2a level may further increase the cytosolic Ca^2+^ level. Our data show that this vicious cycle of an increase in the cytosolic Ca^2+^ level and a decrease in the SERCA2a level can be interrupted by targeting PP1. By targeting PPI with ψPLB-SE, the increased SERCA2a activity may further decrease the cytosolic Ca^2+^ level, which then upregulates the SERCA2a level by attenuating the calpain-mediated SERCA2a degradation.

Taken together, we demonstrated that targeting PP1 with ψPLB-SE restored both the level and activity of SERCA2a in VSMC under synthetic conditions. ψPLB-SE may form the basis of a therapeutic strategy for the prevention of vascular proliferative disorders. Peptides may also have other advantages over gene therapies.

## Supporting Information

S1 Fig ψPLB-SE attenuates VSMC migration. (**A**) RASMC were cultured in contractile or synthetic media and scratched. Control or ψPLB-SE peptide was added and further culture for 24 h. The relative distance of cell migration was measured under a phase contrast microscope. Red lines indicate the boundaries of the RASMC cultures. Representative images are shown (left), and cell migration distances are plotted (right) (n = 4). Scale bar, 50 μm. (**B**) RASMC were cultured in contractile media, and synthetic phenotypes were induced by addition of PDGF-BB in the presence of 3 μM of control or ψPLB-SE peptide. Immunostaining was performed with an antibody against PCNA (red). Nuclei were stained with Hoechst (blue). Representative merged images are shown. Scale bar, 50μm. (**C**) Cell proliferation was quantified using a cell viability assay kit (n = 5). (**D**) RASMC were subjected to the scratch assay under the same conditions as described in panel B. Representative images are shown (left), and cell migration distances are plotted (right) (n = 4). Scale bar, 50 μm.
(EPS)

S2 Fig ψPLB-SE attenuates SERCA2a degradation. RASMC were cultured in contractile or synthetic media in the presence of 3 μM of control or ψPLB-SE peptide. Cycloheximide was added to media to a final concentration of 5 μg/ml to prevent *de novo* protein synthesis. Cells were harvested after 0, 3, and 5 days of incubation and their protein extracts were subjected to western blotting. Data are reported as the means ± SD (n = 3–4; *, *P* < 0.05).
(EPS)

S3 Fig ψPLB-SE attenuates PLB dephosphorylation in VSMC. The proteins samples shown in Fig 2C were subjected to western blotting. Antibodies for PLB or phospho-PLB was used. Data are reported as the means ± SD (n = 3–4; *, *P* < 0.05).
(EPS)
